# Some Extended Results of Common Fixed Point Theorems via Enhanced Categories of Contractive Mappings in Dd* - Symmetric Spaces

**DOI:** 10.12688/f1000research.172242.2

**Published:** 2026-02-07

**Authors:** Abduljabar K. Abbas, Ali A. Shihab, Alaa M.F. Al-Jumaili

**Affiliations:** 1 Department of mathematics, College of Computer science and Mathematics, University of Tikrit, Tikrit, Salah Al-Deen, 34001, Iraq; 2 Department of mathematics, College of Education for pure science, University of Tikrit, Tikrit, Salah Al-Deen, 34001, Iraq; 3 Department of mathematics, College of Education for pure sciences, University of Anbar, Ramadi, Anbar, 31001, Iraq

**Keywords:** D*-metric spaces; D_d*-symmetric spaces; common fixed points; (CLR) property; weakly compatibility; (O.W.C) maps;(〖CLR〗_((FF, GJ))) property; uniqueness of common fixed points.

## Abstract

**Background:**

After witnessing the implementations of Banach fixed point theory which is stated that a mapping T: X→X has always a unique fixed point in X in giving the existence and uniqueness solutions for many integral and differential equations, various extensions of Banach fixed point theory were established. Consequently, the theory has evolved to encompass diverse extensions and is fruitful in many fields. One of the most significant advances in pure and applied mathematics is the discovery of solutions for linear and nonlinear systems as well fractal graphics, optimization theory, approximation theory, discrete dynamics and numerous other areas. Our main outcomes in this manuscript represent one of the most important of these extensions.

**Methods and Results:**

Vital concepts such as $D_{d}^{*}$ -Symmetric spaces and weakly compatible maps are reviewed to establish the framework for our main results. The major objective of the present study is to investigate and verify the uniqueness of some common fixed point theorems for three pairs of self-maps under the influence of other enhanced categories of extended contractive conditions in the context of $\;D_{d}^{*}$ -Symmetric spaces. Our first main outcomes were established by applying the concepts of weak compatibility and common limit in the range property, whereas we obtained our second major results by utilizing the notion of occasionally weakly compatible mapping. Additionally, various common fixed point outcomes for the two pairs of self-maps were determined.

**Conclusion:**

This manuscript explores novel outcomes regarding the uniqueness of various common fixed point theorems for three pairs of self-maps under the influence of other enhanced types of extended contractive conditions in the context of $\;D_{d}^{*}$ -Symmetric spaces. We anticipate that the discoveries in this manuscript will aid scientists in enhancing the authors on popularized extended symmetric-spaces to elevate a universal framework for their practical implementations in each advanced branches of science.

## 1. Introduction

The common fixed point theory under influence various classes of extended contractive conditions has been developed over the decades to include various extensions and productive implementations in many fields of mathematics and other branches of science, such as engineering, physics, computer sciences, economics, and telecommunication optimization problems, making it a cornerstone of mathematical analysis and topological spaces. In 1976, Jungck ^1^ extended the celebrated Banach contraction principle by exploiting the idea of commuting maps and established a common fixed point theory. Subsequently, in 1982, Sessa ^2^ started the tradition of modifying commutativity in fixed point theorems by offering the idea of weakly commuting maps. As an extension of commuting maps, the idea of compatible maps was presented by Jungck in 1986, ^3^ which has been frequently applied to verify the existence of common fixed point theorems. In 2002, Aamri and El Moutawakil ^4^ introduced (E-A) property for pairs of self-maps, which is a true extension of non-compatible maps under contractive concisions. Consequently, Liu et al. ^5^ introduced the concept of the common (E-A) property, which contains (E-A) property presented. ^4^ In addition, Jungck and Rhoades ^6^ defined the idea of occasionally weakly compatible maps, which is more general among commutativity ideas, and obtained various common fixed point theorems. Numerous researchers have presented different extensions of the concept of metric spaces. In particular, Shaban et al. in 2007 ^7^ defined the context of $D^{*}$ -metric spaces. Cho et al. ^8^ verified a number of common fixed point theorems for weakly compatible maps and presented various counter examples. Chandra and Bhatt ^9^ confirmed the fixed point theory for extended contraction under restrictive conditions. On the other hand, W. Sintunavarat, ^10^ presented the idea of common limit in the range $\left({CLR}_{\xi }\right)$ property for pair of self-maps in metric spacers. Later, in 2013, Karapinar et al. ^11^ expanded the idea $\left({CLR}_{\xi }\right)$ for two couples of self-maps in symmetric spaces. In addition, Eke ^12^ proved various common fixed point theorems for contraction maps in uniform space. The fixed point theory has expanded rapidly in extended metric spaces talented with partial ordering. Jumaili ^13^ applied $D^{*}$ -metric space and offered various coincidence fixed point theorems for maps satisfying contractive conditions in partially ordered complete $D^{*}$ -metric spaces. Recently (2019), Al-Jumaili et al. ^14^ extended $D^{*}$ -metric-sp by modifying ℝ via an ordered Banach space. Additionally, in 2020, Latif and Abed ^15^ studied fixed point of set-valued contractions in ordered G-metric spaces. In addition, Nagaraju ^16^ defined the idea of weakly contractive maps and proved several common fixed point theorems for three pairs of self-maps in G-metric spaces. As promised studies, we could generalize our outcomes to other spaces, such as. ^17– 23^ Motivated by above facts, N. A. Majid et al. in 2023 ^24^ verified various original fixed point outcomes for monotone multi-valued maps in partially ordered $D^{*}$ -metric spaces, and investigated various existence and uniqueness of coupled fixed point outcomes of maps satisfying contractive conditions, additionally ^25^ they investigated and proved various outcomes of common and coincidence of fixed points in *S*-metric spaces. Subsequently, in 2024, Abed and Al-Jumaili ^26^ defined a novel type of extended metric space, namely $D_{d}^{*}$ -Symmetric space, and established some common fixed point results for maps satisfying extended contractive conditions in $D_{d}^{*}$ -Symmetric spaces.

Recent developments in 2025 have further expanded fixed point theory into complex generalized spaces with robust applications. For instance, new results in $m_{v}^{b}$ -metric spaces have addressed multivalued integral contractions “ https://doi.org/10.15672/hujms.1471688” and Fredholm integral inclusions “ https://doi.org/10.3934/math.2025926”. Furthermore, significant advances have been made in the application of fuzzy metric spaces to nonlinear differential inclusions “ https://doi.org/10.1177/18758967251366331” and proximal contractions to optimization problems “ https://doi.org/10.1186/s13663-025-00799-0”. These works highlight the continued relevance of extending contractive conditions in generalized spaces.

The major goal of the present manuscript is to discuss and verify various common fixed-point theorems for several categories of self-maps under influence of other extended weakly contractive conditions in the context of $\;D_{d}^{*}$ -Symmetric spaces. Additionally, employing the notions of weak compatibility and common limit in the range property, our first major outcomes have been verified, while other major results utilizing the idea of occasionally weakly compatible maps have been obtained. Lastly, our major outcomes, which are related to these categories of common fixed-point theorems for numerous kinds of single-valued maps, improve and extend various recognized analogous outcomes in the literature.

## 2. Materials and methods

This section presents the various definitions and motivations that are needed in the sequel, which will help us in the outcomes that follow and play a major role in verifying our major outcomes.

Definition 2.1: ^26^ A; $D_{d}^{*}$ -Symmetric on X≠∅ is a map $\;D_{d}^{*}:X\times X\times X\to \left[0,\infty \right)$ (s. t) $\forall x^{*},y^{*},z^{*}\in X$ , the next axioms are satisfied:

$$\begin{array}{c} \left(D_{d_{1}}^{*}\right)\;D_{d}^{*}\;\left(x^{*},y^{*},z^{*}\right)\geq 0,\forall x^{*},y^{*},z^{*}\in X;\\ \left(D_{d_{2}}^{*}\right)\;D_{d}^{*}\left(x^{*},y^{*},z^{*}\right)=0\;\mathrm{iff}\;x^{*}=y^{*}=z^{*};\\ \left(D_{d_{3}}^{*}\right)\;D_{d}^{*}\left(x^{*},y^{*},z^{*}\right)=D_{d}^{*}\left(P\left\{x^{*},y^{*},z^{*}\right\}\right),\left(\text{symmetry}\right)\;\left(s.t\right)\;P\;\text{is}\;a\;\text{permutation}\;\mathrm{map}. \end{array}$$

In this case, $\;D_{d}^{*}$ is called $\;D_{d}^{*}$ -Symmetric and $\left(X,D_{d}^{*}\right)$ is called symmetric space.

Example 2.2: ^26^ Presume that X=[0,1] equipped with $D^{*}$ -Symmetric map described through $D_{d}^{*}\left(x^{*},y^{*},z^{*}\right)={\left(x^{*}-y^{*}\right)}^{2}+{\left(y^{*}-z^{*}\right)}^{2}+{\left(z^{*}-x^{*}\right)}^{2},\forall x^{*},y^{*},z^{*}\in X$ . Here, $\left(D_{d}^{*},X\right)$ are $\;D_{d}^{*}$ -Symmetric space.

Definition 2.3: ^26^ Assume that $\left(X,D_{d}^{*}\right)$ is a $\;D_{d}^{*}$ -Symmetric space, therefore a sequence $\left\{x_{s}^{*}\right\}$ is called $D_{d}^{*}$ -converges to $x^{*}\in X$ *iff* $D_{d}^{*}\left(x_{s}^{*},x_{s}^{*},x^{*}\right)=D_{d}^{*}\left(x^{*},x^{*},x_{s}^{*}\right)⟶0\;\mathrm{as}\;s⟶\infty .$

Remark 2.4: Equivalent to Wilson axiom’s ^27^ in $\;D_{d}^{*}$ -Symmetric space as

$\left(W_{1}\right)$ Given $\left\{x_{s}^{*}\right\}$ , $x^{*},y^{*}\in X,D_{d}^{*}\left(x_{s}^{*},x^{*},x^{*}\right)⟶0\;$ with $D_{d}^{*}\left(x_{s}^{*},y^{*},y^{*}\right)⟶0⟹x^{*}=y^{*}.$

$\left(W_{2}\right)$ Given $\left\{x_{s}^{*}\right\}\;$ & $\left\{y_{s}^{*}\right\}$ , such that $x^{*},y^{*}\in X,D_{d}^{*}\left(x_{s}^{*},x^{*},x^{*}\right)⟶0\;$ with $D_{d}^{*}\left(x_{s}^{*},y_{s}^{*},y_{s}^{*}\right)⟶0⟹$ $D_{d}^{*}\left(y_{s}^{*},x^{*},x^{*}\right)⟶0$ .

$\left(W_{3}\right)$ Assume that $\left(X,D_{d}^{*}\right)$ are complete, $\;D_{d}^{*}$ -Symmetric space. For an arbitrary sequence $\left\{x_{s}^{*}\right\}\;\text{in}\;X$ , we have $\lim_{s,r\to \infty }D_{d}^{*}\left(x_{s}^{*},x_{r}^{*},x_{r}^{*}\right)=0$ *, iff* $\lim_{s\to \infty }D_{d}^{*}\left(x_{s}^{*},x_{s+1}^{*},x_{s+1}^{*}\right)=0$ .

The next Lemma is analogue of Lemma ^27^ in $\left(X,D_{d}^{*}\right)$ :

Lemma 2.5: Each $\;D_{d}^{*}$ -Symmetric $\left(X,D_{d}^{*}\right)$ , describes a symmetric $d_{D_{d}^{*}}$ on X via:

$d_{D_{d}^{*}}\left(x^{*},y^{*}\right)=D_{d}^{*}\left(x^{*},y^{*},y^{*}\right)+D_{d}^{*}\left(y^{*},x^{*},x^{*}\right),\forall x^{*},y^{*}\in X$ , that is:

$d_{D_{d}^{*}}\left(x^{*},y^{*}\right)=2D_{d}^{*}\left(x^{*},y^{*},y^{*}\right),\forall x^{*},y^{*}\in X.$

Definition 2.6: ^28^ Assume that F,G : $\left(X,D_{d}^{*}\right)⟶\left(X,D_{d}^{*}\right)$ are single-valued self-maps If $F\left(x^{*}\right)=G\left(x^{*}\right)=w^{*}$ for $x^{*}\in X$ , thus $x^{*}$ is said to be a coincidence point of F & G , and $w^{*}$ is the point of coincidence of F and G .

Definition 2.7: ^29^ Assume that $F,G:\left(X,D_{d}^{*}\right)\to \left(X,D_{d}^{*}\right)$ are single-valued self-maps. *If* $F\left(x^{*}\right)=G\left(x^{*}\right)=x^{*}\;$ for $x^{*}\in X,$ consequently $x^{*}$ is called the common fixed point (C.F.P) of F and G .

Definition 2.8: ^26^ A pair (F,G) of self-maps of $D_{d}^{*}$ -Symmetric $\left(X,D_{d}^{*}\right)$ are called weakly compatible maps (Concisely, W.C.M) if they are commute at their coincidence points.

Definition 2.9: ^6^ Self-maps F and G of $\left(X,D_{d}^{*}\right)$ are called occasionally weakly compatible *iff* ∃x∈X which is coincidence point of F and G which F and G are commute.

Remark 2.10: It is clear that each pair of weakly compatible maps is (O.W.C) map, other than the opposite, and not generally correct.

Definition 2.11: ^30^ μ:[0,∞)→[0,∞) is said to be an altering distance if μ is continuous and nondecreasing with μ(t)=0 *iff* t=0 .

Definition 2.12: ^10^ Two self-maps η and ξ of (X,d) satisfy the property of the common limit in the range of ξ , indicated via $\left({CLR}_{\xi }\right)$ , if $\exists \left\{x_{s}^{*}\right\}$ in X (s. t) $\lim_{s\to \infty }\eta x_{s}^{*}=\lim_{s\to \infty }\xi x_{s}^{*}=\xi p$ for p∈X .

Definition 2.13: ^11^ The pairs (ξ,η) and (F,G) of self-maps in Symmetric space (X,d) are called gratify property of common limit range related to the maps η and G , indicated via $\left({CLR}_{\left(\eta ,G\right)}\right)$ , if $\exists \left\{x_{s}^{*}\right\}$ and $\left\{y_{s}^{*}\right\}$ (s. t) $\lim_{s\to \infty }\xi x_{s}^{*}=\lim_{s\to \infty }\eta x_{s}^{*}=\lim_{s\to \infty }Fy_{s}^{*}=\lim_{s\to \infty }Gy_{s}^{*}=t$ with t=ηp=Gq for some t,p,q∈X .

Remark 2.14: ^10^ If ξ=F and η=G , in that case Definition-2.12 reduces to $\left({CLR}_{\eta }\right)$ property.

## 3. Main results of new extended categories of common fixed point theorems

In this section, we establish the uniqueness of various common fixed-point theorems for three pairs of self-maps satisfying $\left({CLR}_{\left(FF,\mathrm{GJ}\right)}\right)$ properties under influence of other enhanced categories of extended contractive maps in $D_{d}^{*}$ -Symmetric space. Additionally, various common fixed-point outcomes for two pairs of self-maps were identified.

Remark 3.1: Throughout this manuscript, assume that the next properties are hold:

(i) Let $\mathbb{C}=\left\{\begin{array}{c} \psi /\psi :\left[0,\infty \right)\to \left[0,\infty \right)\;\text{is lower semi continuous and}\;\\ \text{nondecreasing}\;\mathrm{map}\;\text{where}\;\psi \left(t\right)=0⟺t=0 \end{array}\right\}$

(ii) Assume that; S,ℚ,F,F,G and J are three pairs of self-maps of a $D_{d}^{*}$ -Symmetric $\left(X,D_{d}^{*}\right)$ , where

$$\mu \left(\;D_{d}^{*}\left(Sx^{*},\mathbb{Q}y^{*},\mathbb{Q}z^{*}\right)\right)\leq \mu \left(L\left(x^{*},y^{*},z^{*}\right)\right)-\psi \left(L\left(x^{*},y^{*},z^{*}\right)\right),\forall x^{*},y^{*},z^{*}\in X$$ (1)

Wherever, μ is altering distance map, ψ∈ℂ with

$$L\left(x^{*},y^{*},z^{*}\right)=\max\left\{\begin{array}{c} D_{d}^{*}\left(FFx^{*},\mathrm{GJ}y^{*},\mathrm{GJ}z^{*}\right),D_{d}^{*}\left(FFx^{*},FFx^{*},Sx^{*}\right),D_{d}^{*}\left(\mathrm{GJ}y^{*},\mathbb{Q}y^{*},\mathbb{Q}z^{*}\right),\\ \frac{1}{3}\;\left\{D_{d}^{*}\left(FFx^{*},\mathbb{Q}y^{*},\mathbb{Q}z^{*}\right)+D_{d}^{*}\left(\mathrm{GJ}y^{*},\mathrm{GJ}y^{*},Sx^{*}\right)\right\} \end{array}\right\}$$

Theorem 3.2: Let S,ℚ,F,F,GandJ are three pairs of self-maps of a $D_{d}^{*}$ -Symmetric space $\left(X,D_{d}^{*}\right)$ satisfy inequality (1) and the following conditions:

(i) If (S,FF) and (ℚ,GJ) gratify ${CLR}_{\left(FF,\mathrm{GJ}\right)}$ property.

(ii) If (S,FF) and (ℚ,GJ) are weakly compatible maps.

In this case, the maps S,ℚ,FF and GJ have a unique (C. F. P) in X . Additionally, S,ℚ,F,F,G and J contain a unique(C. F. P) provided that the pairs of maps (F,F),(S,F),(S,F),(G,J),(ℚ,G) and (ℚ,J) are commuting.

Proof:
Because (S,FF) and (ℚ,GJ) satisfy $\left({CLR}_{\left(FF,\mathrm{GJ}\right)}\right)$ property, we can discover two sequences $\left\{x_{s}^{*}\right\}$ and $\left\{y_{s}^{*}\right\}$ in X (s. t), $\lim_{s\to \infty }Sx_{s}^{*}=\lim_{s\to \infty }FFx_{s}^{*}=\lim_{s\to \infty }\mathbb{Q}y_{s}^{*}=\lim_{s\to \infty }\mathrm{GJ}y_{s}^{*}=t$ with t=FFp=GJq for some t,p,q∈X .

**Firstly** verify FFp=Sp . From inequality (1) and $x^{*}=p,y^{*}=z^{*}=y_{s}^{*}$ , we get

$$\mu \left(\;D_{d}^{*}\left(Sp,Sp,\mathbb{Q}y^{*}\right)\right)\leq \mu \left(L\left(p,p,y_{s}^{*}\right)\right)-\psi \left(L\left(p,p,y_{s}^{*}\right)\right)$$ (2)

Such that

$$L\left(p,p,y_{s}^{*}\right)=\max\left\{\begin{array}{c} D_{d}^{*}\left(F\mathrm{Fp},F\mathrm{Fp},\mathrm{GJ}y_{s}^{*}\right),D_{d}^{*}\left(F\mathrm{Fp},F\mathrm{Fp},Sp\right),D_{d}^{*}\left(\mathrm{GJ}y_{s}^{*},\mathrm{GJ}y_{s}^{*},\mathbb{Q}y_{s}^{*}\right),\\ \frac{1}{3}\left\{\;D_{d}^{*}\left(F\mathrm{Fp},F\mathrm{Fp},\mathbb{Q}y_{s}^{*}\right)+D_{d}^{*}\left(\mathrm{GJ}y_{s}^{*},\mathrm{GJ}y_{s}^{*},Sp\right)\right\} \end{array}\right\}$$

Consequently

$$\lim_{s\to \infty }L\left(p,p,y_{s}^{*}\right)=\max\left\{\begin{array}{c} \;D_{d}^{*}\left(t,t,t\right),D^{*}\left(t,t,Sp\right),D_{d}^{*}\left(t,t,t\right),\\ \frac{1}{3}\left\{\;D_{d}^{*}\left(t,t,t\right)+D_{d}^{*}\left(t,t,Sp\right)\right\} \end{array}\right\}$$

$$=\max\left\{0,D_{d}^{*}\left(t,t,Sp\right),0,\frac{1}{3}\left\{\;D_{d}^{*}\left(t,t,Sp\right)\right\}\right\}=D_{d}^{*}\left(Sp,Sp,t\right),\;\text{by using}\left(D_{d_{3}}^{*}\right)\text{of Definition 2.1}.$$

Let s→∞ in inequality (2), $\mu \left(\;D_{d}^{*}\left(Sp,Sp,t\right)\right)\leq \mu \left(\;D_{d}^{*}\left(Sp,Sp,t\right)\right)-\psi \left(D_{d}^{*}\left(Sp,Sp,t\right)\right)$ which implies $\psi \left(\;D_{d}^{*}\left(Sp,Sp,t\right)\right)=0$ , as a result $\;D_{d}^{*}\left(Sp,Sp,t\right)=0$ , that is, Sp=t=FFp , illustrating p is the coincidence point of S and FF .

Because (S,FF) is weakly compatible, we have S(FF)p=(FF)Sp , therefore St=FFt .

**Secondly** verify: ℚq=GJq . From inequality (1) (s. t), $x^{*}=x_{s}^{*},y^{*}=z^{*}=q$ , get that

$$\mu \left(\;D_{d}^{*}\left(Sx_{s}^{*},Sx_{s}^{*},\mathbb{Q}q\right)\right)\leq \mu \left(L\left(x_{s}^{*},x_{s}^{*},q\right)\right)-\psi \left(L\left(x_{s}^{*},x_{s}^{*},q\right)\right)$$ (3)

Where

$$L\left(x_{s}^{*},x_{s}^{*},q\right)=\max\left\{\begin{array}{c} D_{d}^{*}\left(FFx_{s}^{*},FFx_{s}^{*},\mathrm{GJq}\right),D_{d}^{*}\left(FFx_{s}^{*},FFx_{s}^{*},Sx_{s}^{*}\right),D_{d}^{*}\left(\mathrm{GJq},\mathrm{GJq},\mathbb{Q}q\right),\\ \frac{1}{3}\left\{\;D_{d}^{*}\left(FFx_{s}^{*},FFx_{s}^{*},\mathbb{Q}q\right)+D_{d}^{*}\left(\mathrm{GJq},\mathrm{GJq},Sx_{s}^{*}\right)\right\} \end{array}\right\}$$

Therefore,

$$\begin{array}{c} \lim_{s\to \infty }L\left(x_{s}^{*},x_{s}^{*},q\right)=\max\left\{\begin{array}{c} D_{d}^{*}\left(t,t,t\right),D^{*}\left(t,t,t\right),D_{d}^{*}\left(t,t,\mathbb{Q}q\right),\\ \frac{1}{3}\left\{\;D_{d}^{*}\left(t,t,\mathbb{Q}q\right)+D_{d}^{*}\left(t,t,t\right)\right\} \end{array}\right\}\\ =\max\left\{0,0,D^{*}\left(t,t,\mathbb{Q}q\right),\frac{1}{3}\left\{D_{d}^{*}\left(t,t,\mathbb{Q}q\right)+0\right\}\right\}=D_{d}^{*}\left(t,t,\mathbb{Q}q\right). \end{array}$$

Selecting limit as s→∞ in inequality (3),

$$\mu \left(D_{d}^{*}\left(t,t,\mathbb{Q}q\right)\right)\leq \mu \left(D_{d}^{*}\left(t,t,\mathbb{Q}q\right)\right)-\psi \left(D_{d}^{*}\left(t,t,\mathbb{Q}q\right)\right)$$

This implies that $\left(\;D_{d}^{*}\left(t,t,\mathbb{Q}q\right)\right)=0$ , as a result $\;D_{d}^{*}\left(t,t,\mathbb{Q}q\right)=0$ ,that is, ℚq=t=GJq , explaining q is the coincidence point of ℚ and GJ . As (ℚ,GJ) is (W.C.M), we obtain ℚ(GJ)q=(GJ)ℚq , and consequently ℚt=GJt .

**Third** verify: St=FFt=t . From inequality (1) (s. t) $x^{*}=t,y^{*}=z^{*}=q$ , obtain

$$\begin{array}{c} \mu \left(D_{d}^{*}\left(St,St,\mathbb{Q}q\right)\right)\leq \mu \left(L\left(t,t,q\right)\right)-\psi \left(L\left(t,t,q\right)\right)\\ \text{Or}\mu \left(D_{d}^{*}\left(St,St,t\right)\right)\leq \mu \left(L\left(t,t,q\right)\right)-\psi \left(L\left(t,t,q\right)\right) \end{array}$$

Such that

$$L\left(t,t,q\right)=\max\left\{\begin{array}{c} D_{d}^{*}\left(F\text{Ft},F\text{Ft},\mathrm{GJq}\right),D_{d}^{*}\left(F\text{Ft},F\text{Ft},St\right),D_{d}^{*}\left(\mathrm{GJq},\mathrm{GJq},\mathbb{Q}q\right),\\ \frac{1}{3}\left\{\;D_{d}^{*}\left(F\text{Ft},F\text{Ft},\mathbb{Q}q\right)+D_{d}^{*}\left(\mathrm{GJq},\mathrm{GJq},St\right)\right\} \end{array}\right\}$$

Consequently

$$\begin{array}{c} \max\left\{D_{d}^{*}\left(St,St,t\right),D_{d}^{*}\left(St,St,St\right),D_{d}^{*}\left(t,t,t\right),\frac{1}{3}\left\{\;D_{d}^{*}\left(St,St,t\right)+D_{d}^{*}\left(t,t,St\right)\right\}\right\}\\ =\max\left\{D_{d}^{*}\left(St,St,t\right),0,0,\frac{2}{3}\left\{\;D_{d}^{*}\left(St,St,t\right)\right\}\right\},\;\text{by using}\left(D_{d_{3}}^{*}\right)\text{of Definition 2.1}.\\ =D_{d}^{*}\left(St,St,t\right). \end{array}$$

Thus, $\mu \left(D_{d}^{*}\left(St,St,t\right)\right)\leq \mu \left(D_{d}^{*}\left(St,St,t\right)\right)-\psi \left(D_{d}^{*}\left(St,St,t\right)\right)⟹$ $\psi \left(\;D_{d}^{*}\left(St,St,t\right)\right)=0$ , as a result $D^{*}\left(St,St,t\right)=0$ , that is, St=t . Consequently, St=FFt=t .

**Finally** verify: ℚt=GJt=t. From inequality- (1) (s. t) $x^{*}=p,y^{*}=z^{*}=t$ , we obtain

$$\begin{array}{c} \mu \left(\;D_{d}^{*}\left(Sp,Sp,\mathbb{Q}t\right)\right)\leq \mu \left(L\left(p,p,t\right)\right)-\psi \left(L\left(p,p,t\right)\right)\\ \mathrm{Or\mu}\left(D_{d}^{*}\left(t,t,\mathbb{Q}t\right)\right)\leq \mu \left(L\left(p,p,t\right)\right)-\psi \left(L\left(p,p,t\right)\right) \end{array}$$

Such that

$$\begin{array}{c} L\left(p,p,t\right)=\max\left\{\begin{array}{c} D_{d}^{*}\left(F\mathrm{Fp},F\mathrm{Fp},\mathrm{GJt}\right),D_{d}^{*}\left(F\mathrm{Fp},F\mathrm{Fp},Sp\right),D_{d}^{*}\left(\mathrm{GJt},\mathrm{GJt},\mathbb{Q}t\right),\\ \frac{1}{3}\left\{\;D_{d}^{*}\left(F\mathrm{Fp},F\mathrm{Fp},\mathbb{Q}t\right)+D_{d}^{*}\left(\mathrm{GJt},\mathrm{GJt},Sp\right)\right\} \end{array}\right\}\\ =\max\left\{D_{d}^{*}\left(t,t,\mathbb{Q}t\right),D_{d}^{*}\left(t,t,t\right),D^{*}\left(\mathbb{Q}t,\mathbb{Q}t,\mathbb{Q}t\right),\frac{1}{3}\left\{\;D_{d}^{*}\left(t,t,\mathbb{Q}t\right)+D_{d}^{*}\left(\mathbb{Q}t,\mathbb{Q}t,t\right)\right\}\right\}\\ \begin{array}{c} =\max\left\{D_{d}^{*}\left(t,t,\mathbb{Q}t\right),0,0,\frac{1}{3}\left\{D_{d}^{*}\left(t,t,\mathbb{Q}t\right)+D_{d}^{*}\left(\mathbb{Q}t,\mathbb{Q}t,t\right)\right\}\right\}\\ =\max\left\{D_{d}^{*}\left(t,t,\mathbb{Q}t\right),\frac{2}{3}\left\{D_{d}^{*}\left(t,t,\mathbb{Q}t\right)\right\}\right\},\;\text{by using}\left(D_{d_{3}}^{*}\right)\text{of Definition 2.1}.\\ =D_{d}^{*}\left(t,t,\mathbb{Q}t\right). \end{array} \end{array}$$

Consequently, $\mu \left(D_{d}^{*}\left(t,t,\mathbb{Q}t\right)\right)\leq \mu \left(D_{d}^{*}\left(t,t,\mathbb{Q}t\right)\right)-\psi \left(D_{d}^{*}\left(t,t,\mathbb{Q}t\right)\right)⟹\left(D_{d}^{*}\left(t,t,\mathbb{Q}t\right)\right)=0.$ Therefore $\;D_{d}^{*}\left(t,t,\mathbb{Q}t\right)=0$ , that is, ℚt=t . Consequently, ℚt=GJt=t , thus St=FFt=ℚt=GJt=t , demonstrating t is (C. F. P) of S,ℚ,FF and JG .

***Uniqueness:*** Assume that (n≠t) is different (C. F. P) for S,ℚ,FF and JG . Consequently, we have Sn=FFn=ℚn=GJn=n . Now, from inequality (1) with $x^{*}=t,y^{*}=z^{*}=n$ , obtain

$$\begin{array}{c} \mu \left(D_{d}^{*}\left(St,St,\mathbb{Q}n\right)\right)\leq \mu \left(L\left(t,t,n\right)\right)-\psi \left(L\left(t,t,n\right)\right)\\ \text{Or}\mu \left(D_{d}^{*}\left(t,t,n\right)\right)\leq \mu \left(L\left(t,t,n\right)\right)-\psi \left(L\left(t,t,n\right)\right), \end{array}$$

Such that

$$\begin{array}{c} L\left(t,t,n\right)=\max\left\{\begin{array}{c} D_{d}^{*}\left(F\text{Ft},F\text{Ft},\mathrm{GJn}\right),D_{d}^{*}\left(F\text{Ft},F\text{Ft},St\right),D_{d}^{*}\left(\mathrm{GJn},\mathrm{GJn},\mathbb{Q}n\right),\\ \frac{1}{3}\left\{D_{d}^{*}\left(F\text{Ft},F\text{Ft},\mathbb{Q}n\right)+D_{d}^{*}\left(\mathrm{GJn},\mathrm{GJn},St\right)\right\} \end{array}\right\}\\ =\max\left\{\;D_{d}^{*}\left(t,t,n\right),D_{d}^{*}\left(t,t,t\right),D_{d}^{*}\left(n,n,n\right),\frac{1}{3}\left\{D_{d}^{*}\left(t,t,n\right)+D_{d}^{*}\left(n,n,t\right)\right\}\right\}\\ \begin{array}{c} =\max\left\{D_{d}^{*}\left(t,t,n\right),0,0,\frac{1}{3}\left\{\;D_{d}^{*}\left(t,t,n\right)+D_{d}^{*}\left(n,n,t\right)\right\}\right\}\\ =\max\left\{\;D_{d}^{*}\left(t,t,n\right),\frac{2}{3}\left\{D_{d}^{*}\left(t,t,n\right)\right\}\right\}=D_{d}^{*}\left(t,t,n\right),\;\text{by using}\left(D_{d_{3}}^{*}\right)\text{of Definition 2.1}. \end{array} \end{array}$$

Therefore,

$$\mu \left(D_{d}^{*}\left(t,t,n\right)\right)\leq \mu \left(D_{d}^{*}\left(t,t,n\right)\right)-\psi \left(D_{d}^{*}\left(t,t,n\right)\right)⟹\psi \left(D_{d}^{*}\left(t,t,n\right)\right)=0.$$

Consequently, $\;D_{d}^{*}\left(t,t,n\right)=0$ , that is, t=n . Thus, S,FF,ℚ,JG have unique common fixed point in X .

Next, we shall verify S,ℚ,F,F,G and J have unique common fixed point.

Because, (F,F),(S,F),(S,F) are commuting, so Ft=F(FFt)=(FF)Ft with Ft=F(St)=S(Ft) .

In addition, Ft=F(FFt)=(FF)Ft with Ft=F(St)=S(Ft) .

This illustrates Ft and Ft are (C. F. Ps) of FF and S . Therefore, via the uniqueness of common fixed point, we obtain Ft=Ft=t .

Likewise, because (G,J),(ℚ,G) and (ℚ,J) are commuting, obtain Gt=G(ℚt)=(ℚG)t with Gt=G(GJt)=GJ(Gt) .

Moreover, Jt=J(ℚt)=ℚ(Jt) and Jt=J(GJt)=GJ(Jt) . This explains Gt and Jt are common fixed point of GJ and ℚ . Consequently, via the uniqueness of common fixed point, obtain Gt=Jt=t . As a result, St=ℚt=Ft=Ft=Gt=Jt=t , verifying t is unique common fixed point to S,ℚ,F,F,G and J.

Corollary 3.3: If S,ℚ,F and J are two pairs of self-maps of $\;D_{d}^{*}$ -Symmetric $\left(X,D_{d}^{*}\right)$ satisfying the following conditions:

(i) If $\mu \left(D_{d}^{*}\left(Sx^{*},\mathbb{Q}y^{*},\mathbb{Q}z^{*}\right)\right)\leq \mu \left(L\left(x^{*},y^{*},z^{*}\right)\right)-\psi \left(L\left(x^{*},y^{*},z^{*}\right)\right)\forall x^{*},y^{*},z^{*}\in X.$ (s. t), μ alters the distance map with $\psi :{\mathbb{R}}^{+}\to {\mathbb{R}}^{+}$ , is a lower semi-continuous and nondecreasing map (s. t) ψ(t)=0 *iff* t=0 with

$$L\left(x^{*},y^{*},z^{*}\right)=\max\left\{\begin{array}{c} D_{d}^{*}\left(Fx^{*},Jy^{*},Jz^{*}\right),D_{d}^{*}\left(Fx^{*},Fx^{*},Sx^{*}\right),D_{d}^{*}\left(Jy^{*},\mathbb{Q}y^{*},\mathbb{Q}z^{*}\right),\\ \frac{1}{3}\left\{D_{d}^{*}\left(Fx^{*},\mathbb{Q}y^{*},\mathbb{Q}z^{*}\right)+D_{d}^{*}\left(Jy^{*},Jy^{*},Sx^{*}\right)\right\} \end{array}\right\}$$

(ii) If (S,F) and (ℚ,J) satisfy $\left({CLR}_{\left(F,J\right)}\right)$ property.

(iii) If (S,F) and (ℚ,J) are (W. C. M). In this case, S,ℚ,F and J contained unique (C. F. P) in X .

Proof:
Its follows immediately from Theorem-3.2, via putting $G=F=I_{X}$ (Identity map).

Lemma 3.4: Assume that; X≠∅ , with ξ and η are (O. W. C) maps. *If* ξ and η include a unique point of coincidence $w^{*}=\xi t=\eta t$ for t∈X , then $w^{*}$ is unique (C. F. P) of ξ and η .

Theorem 3.5: If S,ℚ,F,F,G and J are three pairs of self-maps in $\;D_{d}^{*}$ -symmetric $\left(X,D_{d}^{*}\right)$ satisfies the following conditions:

(i) If $\mu \left(D_{d}^{*}\left(Sx^{*},\mathbb{Q}y^{*},\mathbb{Q}z^{*}\right)\right)\leq \mu \left(M\left(x^{*},y^{*},z^{*}\right)\right)-\psi \left(M\left(x^{*},y^{*},z^{*}\right)\right),\forall x^{*},y^{*},z^{*}\in X$ , (s. t), μ μ alters the distance map with $\psi :{\mathbb{R}}^{+}\to {\mathbb{R}}^{+}$ , is a lower semi-continuous and nondecreasing map (s. t) ψ(t)=0 *iff* t=0 with

$$M\left(x^{*},y^{*},z^{*}\right)=\max\left\{\begin{array}{c} D_{d}^{*}\left(FFx^{*},\mathrm{GJ}y^{*},\mathrm{GJ}z^{*}\right),D_{d}^{*}\left(FFx^{*},FFx^{*},Sx^{*}\right),\\ D_{d}^{*}\left(\mathrm{GJ}y^{*},\mathbb{Q}y^{*},\mathbb{Q}z^{*}\right),D_{d}^{*}\left(\mathrm{GJ}y^{*},\mathrm{GJ}y^{*},Sx^{*}\right) \end{array}\right\}$$

(ii) If (S,FF) and (ℚ,GJ) are (O. W. C) maps. In this case, the maps S,ℚ,FF and GJ have a unique (C. F. P) in X . Additional S,ℚ,F,F,G and J include unique (C.F.P), provided the pairs of maps (F,F),(S,F),(S,F),(G,J),(ℚ,G) and (ℚ,J) are commuting.

Proof:
Assuming that (S,FF) and (ℚ,GJ) are (O. W. C), we can discover p and q in X (s. t) $\;Sp=F\mathrm{Fp}=p^{1}$ and S(FF)p=(FF)Sp with $\mathbb{Q}q=\mathrm{GJq}=q^{1}$ and ℚ(GJ)q=(GJ)ℚq .

**Firstly,** verify: Sp=ℚq . From condition (i), (s. t) $x^{*}=p,y^{*}=z^{*}=q$ , we get

$$\mu \left(D_{d}^{*}\left(Sp,Sp,\mathbb{Q}q\right)\right)\leq \mu \left(M\left(p,p,q\right)\right)-\psi \left(M\left(p,p,q\right)\right)$$ (4)

Such that

$$\begin{array}{c} M\left(p,p,q\right)=\max\left\{\begin{array}{c} D_{d}^{*}\left(F\mathrm{Fp},F\mathrm{Fp},\mathrm{GJq}\right),D_{d}^{*}\left(F\mathrm{Fp},F\mathrm{Fp},Sp\right),\\ D_{d}^{*}\left(\mathrm{GJq},\mathrm{GJq},\mathbb{Q}q\right),D_{d}^{*}\left(\mathrm{GJq},\mathrm{GJq},Sp\right) \end{array}\right\}\\ \;=\max\left\{\begin{array}{c} D_{d}^{*}\left(Sp,Sp,\mathbb{Q}q\right),D_{d}^{*}\left(Sp,Sp,Sp\right),\\ D_{d}^{*}\left(\mathbb{Q}q,\mathbb{Q}q,\mathbb{Q}q\right),D_{d}^{*}\left(\mathbb{Q}q,\mathbb{Q}q,Sp\right) \end{array}\right\}\\ \begin{array}{c} \;=\max\left\{D_{d}^{*}\left(Sp,Sp,\mathbb{Q}q\right),0,0,D_{d}^{*}\left(\mathbb{Q}q,\mathbb{Q}q,Sp\right)\right\}\\ \;=D_{d}^{*}\left(Sp,Sp,\mathbb{Q}q\right),\;\text{by using}\left(D_{d_{3}}^{*}\right)\text{of Definition 2.1}. \end{array} \end{array}$$

Consequently, $\mu \left(D_{d}^{*}\left(Sp,Sp,\mathbb{Q}q\right)\right)\leq \mu \left(D_{d}^{*}\left(Sp,Sp,\mathbb{Q}q\right)\right)-\psi \left(D_{d}^{*}\left(Sp,Sp,\mathbb{Q}q\right)\right)$ which implies that $\psi \left(D_{d}^{*}\left(Sp,Sp,\mathbb{Q}q\right)\right)=0$ , as a result $D_{d}^{*}\left(Sp,Sp,\mathbb{Q}q\right)=0$ , that is, Sp=ℚq . Therefore, Sp=FFp=ℚq=GJq .

Suppose that $p^{1}$ is another point where ${Sp}^{1}=FFp^{1}$ . In this case, obtain from condition (i), that ${Sp}^{1}=FFp^{1}=\mathbb{Q}q=\mathrm{GJq}$ . Thus $,Sp={Sp}^{1}$ , that is, $p=p^{1}$ , demonstrating S and FF include a unique point of coincidence. Consequently, via Lemma-3.4, get S and FF include unique (C.F.P), namely, t . Likewise, it can be verified ℚ and GJ include unique (C.F.P), say $t^{1}$ .

**Secondly** verify, $t=t^{1}$ . From condition (i), (s. t) $x^{*}=t,y^{*}=z^{*}=t^{1}$ , get

$$\begin{array}{c} \mu \left(D_{d}^{*}\left(St,St,\mathbb{Q}t^{1}\right)\right)\leq \mu \left(M\left(t,t,t^{1}\right)\right)-\psi \left(M\left(t,t,t^{1}\right)\right)\\ \text{Or}\;\mu \left(D_{d}^{*}\left(t,t,t^{1}\right)\right)\leq \mu \left(M\left(t,t,t^{1}\right)\right)-\psi \left(M\left(t,t,t^{1}\right)\right) \end{array}$$ (5)

Where

$$\begin{array}{c} M\left(t,t,t^{1}\right)=\max\left\{\begin{array}{c} D_{d}^{*}\left(F\text{Ft},F\text{Ft},\mathrm{GJ}t^{1}\right),D_{d}^{*}\left(F\text{Ft},F\text{Ft},St\right),\\ D_{d}^{*}\left(\mathrm{GJ}t^{1},\mathrm{GJ}t^{1},\mathbb{Q}t^{1}\right),D_{d}^{*}\left(\mathrm{GJ}t^{1},\mathrm{GJ}t^{1},St\right) \end{array}\right\}\\ =\max\left\{\begin{array}{c} D_{d}^{*}\left(t,t,t^{1}\right),D_{d}^{*}\left(t,t,t\right),\\ D_{d}^{*}\left(t^{1},t^{1},t^{1}\right),D_{d}^{*}\left(t^{1},t^{1},t\right) \end{array}\right\}\\ \begin{array}{c} =\max\left\{D_{d}^{*}\left(t,t^{1},t^{1}\right),0,0,D_{d}^{*}\left(t^{1},t,t\right)\right\}\\ =D_{d}^{*}\left(t,t,t^{1}\right),\;\text{by using}\left(D_{d_{3}}^{*}\right)\text{of Definition 2.1}. \end{array} \end{array}$$

Therefore, $\mu \left(D_{d}^{*}\left(t,t,t^{1}\right)\right)\leq \mu \left(D_{d}^{*}\left(t,t,t^{1}\right)\right)-\psi \left(D_{d}^{*}\left(t,t,t^{1}\right)\right)⟹\psi \left(D_{d}^{*}\left(t,t,t^{1}\right)\right)=0$ , as a result $D_{d}^{*}\left(t,t,t^{1}\right)=0$ , that is, $t=t^{1}$ . Consequently, S,ℚ,FF and GJ are unique (C. F. P). The rest of the evidence is similar to of Theorem-3.2, for this reason, obtain St=ℚt=Ft=Ft=Gt=Jt=t , verifying t is unique(C.F.P) to S,ℚ,F,F,G and J .

Corollary 3.6: If S,ℚ,F and J are two pairs of self-maps of a $\;D_{d}^{*}$ -symmetric $\left(X,D_{d}^{*}\right)$ satisfying the next conditions:

(i) If $\;\mu \left(D_{d}^{*}\left(Sx^{*},\mathbb{Q}y^{*},\mathbb{Q}z^{*}\right)\right)\leq \mu \left(M\left(x^{*},y^{*},z^{*}\right)\right)-\psi \left(M\left(x^{*},y^{*},z^{*}\right)\right),\forall x^{*},y^{*},z^{*}\in X$ , (s. t), μ μ altering distance map with $\psi :{\mathbb{R}}^{+}⟶{\mathbb{R}}^{+}$ , is lower semi-continuous and nondecreasing map (s. t) ψ(t)=0 , *iff* t=0 , with

$$M\left(x^{*},y^{*},z^{*}\right)=\max\left\{\begin{array}{c} D_{d}^{*}\left(Fx^{*},Jy^{*},Jz^{*}\right),D_{d}^{*}\left(Fx^{*},Fx^{*},Sx^{*}\right),\\ D_{d}^{*}\left(Jy^{*},\mathbb{Q}y^{*},\mathbb{Q}z^{*}\right),D_{d}^{*}\left(Jy^{*},Jy^{*},Sx^{*}\right) \end{array}\right\}$$

(ii) If (S,F) and (ℚ,J) are (O. W. C) maps. In this case, maps S,ℚ,F and J have a unique common fixed point in X .

Proof:
This follows directly from Theorem-3.5, by putting $F=G=I_{X}$ (Identity map).

## 4. Conclusion

The study of common fixed point theory for various categories of single-valued mappings under the influence of generalized contractive conditions in extended symmetric-spaces has witnessed a spectacular development of interest in the past few decades. Apparently, it is crucial in numerous disciplines in pure and applied mathematics, and it has various originative implementations in other branches of science. One of the most significant advances in pure and applied mathematics is the discovery of solutions for linear and nonlinear systems as well as fractal graphics, optimization theory, approximation theory, discrete dynamics, and numerous other areas. Therefore, some novel common fixed point theorems for three pairs of self-maps under influence of new extended contractive conditions in the context of $\;D_{d}^{*}$ -Symmetric spaces were investigated and verified. In addition, utilizing the concepts of weak compatibility and a common limit in the range property, our first outcomes are established, whereas (O.W.C) maps are applied to obtain our second outcomes. Our results generalize and improve various recent outcomes of (C.F.P) theorems under extended weakly contractive maps in the literature. We anticipate that these results will aid researchers in developing generalized symmetric spaces and establishing frameworks for applications in advanced sciences.

## Authors’ declaration

### All research studies on humans (individuals, samples)

No human (individuals and samples) studies are present in the manuscript.

## Ethical approval

We would like to inform you that our study does not require any ethical approval.

## Data Availability

No datasets were generated or analyzed during the current study (Our manuscript type does not require data).

## References

[1] Jungck G : Commuting mappings and fixed points. *Am. Math. Mon.* 1976;83:261–263. 10.1080/00029890.1976.11994093

[2] Sessa S : on a weak commutativity condition of mappings in fixed point considerations. *Publ. Inst. Math.* 1982;32:149–153.

[3] Jungck G : Compatible mappings and common fixed points. *Int. J. Math. Math. Sci.* 1986;9:771–779. 10.1155/S0161171286000935

[4] Aamri M El Moutawakil D : some new common fixed point theorems under strict contractive conditions. *J. Math. Anal. Appl.* 2002;270:181–188. 10.1016/S0022-247X(02)00059-8

[5] Liu Y Wu J Li Z : common fixed points of single-valued and multi-valued maps. *Int. J. Math. Math. Sci.* 2005;2005:3045–3055. 10.1155/IJMMS.2005.3045

[6] Jungck G Rhoades BE : fixed point theorems for occasionally weakly compatible mappings. *Fixed Point Theory.* 2006;7(2):287–296.

[7] Shaban S Nabi S Haiyun Z : A common Fixed Point Theorem in D ^*^-Metric Spaces. *Hindawi Publishing Corporation, Fixed Point Theory and Applications.* 2007; p.13. Article ID 27906.

[8] Cho SH Lee GY Bae JS : on coincidence and fixed point theorems in symmetric space. *Fixed Point Theory Appl.* 2008;2008;9. Art.ID 562130. 10.1155/2008/562130

[9] Chandra H Bhatt A : Some fixed point theorems for set valued maps in symmetric spaces. *Int. J. Math. Anal.* 2009;3:839–846.

[10] Sintunavarat W Kumam P : common fixed point theorems for a pair of weakly compatible mappings in fuzzy metric spaces. *J. Appl. Math.* 2011;2011. Article ID 637958. 10.1155/2011/637958

[11] Karapinar E Patel DK Imdad M : Some non unique fixed point theorems in symmetric spaces through CLR(S,T) property. *Int. J. Math. Math. Sci.* 2013;2013;1–8. Article ID 753965. 10.1155/2013/753965

[12] Eke KS : common fixed point theorems for generalized contraction mappings on uniform spaces. *Far East J. Appl. Math.* 2016;99(11):1753–1760. 10.17654/MS099111753

[13] Al Jumaili AMF : Some Coincidence and Fixed Point Results in Partially Ordered Complete Generalized D ^*^- Metric Spaces. *Eur. J. Pure Appl. Math.* 2017;10(5):1024–1035.

[14] Al-Jumaili AM Abed MM Al-sharqi FG : on fixed point theorems and ∇-distance in complete partially ordered G-cone metric spaces. *J. Anal. Appl.* 2019;17(1):1–20.

[15] Latif SQ Abed SS : Types of Fixed Points of Set-Valued Contraction Mappings for Comparable Elements. *Iraqi J. Sci.* 2020;190–195. Special Issue. 10.24996/ijs.2020.SI.1.25

[16] Nagaraju V : Common Fixed Point Theorems for Six Self-Maps in G-Metric Spaces. *Ann. Pure Appl. Math.* 2020;22(1):57–64. 10.22457/apam.v22n1a08690

[17] Mohsen SD : some generalizations of fuzzy soft (k ^∗^–Â)-quasi normal operators in fuzzy soft Hilbert spaces. *J. Interdiscip. Math.* 2023;26(6):1133–1143. 10.47974/JIM-1612

[18] Mohsen SD Thiyab YH : some Characteristics of Completeness Property in Fuzzy Soft b-Metric Space. *J. Appl. Sci. Eng.* 2023;27(3):2227–2232.

[19] Hijab AA Shaakir LK Aljohani S : Fredholm integral equation in composed-cone metric spaces. *Bound. Value Probl.* 2024;2024(64):1–12. 10.1186/s13661-024-01876-w

[20] Hijab AA Shaakir LK : New Generalization of Strong-Composed Metric Type Spaces with Special (ψ,∅)-Contraction. *Adv. Fixed Point Theory.* 2025;15(5):1–20.

[21] Majid NA Al-Jumaili AMF Ng ZC : New Results of Fixed-Point Theorems and Their Applications in Complete Complex D ^∗^ _c_-Metric Spaces. *J. Funct. Spaces* 2024;2024:5532624. 10.1155/2024/5532624

[22] Oklah TN Al-Jumaili AMF : Fixed Point Results of Integral Type Contractive Mappings in D*-Metric Spaces. *AIP Conference Proceedings, 2nd International Conference on Scientific Research and Innovation 2023, 2ICSRI 2023.* 2025;3169(1).

[23] Abed AH Al-Jumaili AMF : On Weakly Compatible Maps and Some Common Fixed Point Theorems in D*-Metric Spaces. *AIP Conference Proceedings, 2nd International Conference on Scientific Research and Innovation 2023, 2ICSRI 2023.* 2025;3169(1).

[24] Majid NA Alaa MF Al-Jumaili ZC : Lee, some applications of fixed point results for monotone multivalued and integral type contractive mappings. *Fixed Point Theory Algorithms Sci. Eng.* 2023;2023(1):1–11. 10.1186/s13663-023-00748-9

[25] Majid NA Al-Jumaili A Ng ZC : Enhanced Results in Common and Coincidence Fixed Point Theory with Applications to Simulation Mappings. *Eur. J. Pure Appl. Math.* 2024;17(3):1877–1893. 10.29020/nybg.ejpam.v17i3.5268

[26] Abed AH Al-Jumaili AMF : Occasionally Weakly Compatible Maps and common Fixed Point Theorems in Certain New Generalized Symmetric Space. *Iraqi J. Sci.* 2024;65(8):4419–4427. 10.24996/ijs.2024.65.8.24

[27] Wilson WA : On Semi-Metric Spaces. *Am. J. Math.* 1931;53(2):361–373. 10.2307/2370790

[28] Abbas M Jungck G : common fixed point results for non-commuting mappings without continuity in cone metric spaces. *J. Math. Anal. Appl.* 2008;341(1):416–420. 10.1016/j.jmaa.2007.09.070

[29] Jungck G : common fixed points for non-continuous non-self maps on non-metric spaces. *Far East J. Math. Sci.* 1996;4(2):199–215.

[30] Khan MS Swaleh M Sessa S : fixed point theorems by altering distance between the points. *Bull. Aust. Math. Soc.* 1984;30:1–9. 10.1017/S0004972700001659

