# Supplementary material for: Some Extended Results of Common Fixed Point Theorems via Enhanced Categories of Contractive Mappings in Dd* - Symmetric Spaces
Source: F1000Res. 2026 Feb 7;14:1363. Originally published 2025 Dec 5. [Version 2] doi: 10.12688/f1000research.172242.2 (PMC13142793; doi:10.12688/f1000research.172242.2)
Supplement: Peer review history

# Reviewer report: Reviewer response for version 2

**DOI:** 10.5256/f1000research.195954.r456794

**Published:** 2026-03-14

**Authors:** Kapil Yadav

**Affiliations:** 1 Lovely Professional University, Phagwara, Punjab, India

**Article Title:**

 Some Extended Results of Common Fixed-Point Theorems via Enhanced Categories of Contractive Mappings in Dd* - Symmetric Spaces

**Article summary:**

 The manuscript establishes common fixed-point results for pairs of contractive mappings satisfying weak compatibility and CLR property using altering distance functions in the setting of Dd*symmetric spaces. In addition, the authors derive common fixed-point results for occasionally weakly compatible mappings in this framework. Several corollaries are also presented as consequences of the main theorem. The results generalize and improve various recent outcomes of (C.F.P) theorems under extended weakly contractive maps in the literature. The proofs of the theorems are well illustrated and are presented in a clear and understandable manner. In order to enhance the readability of the manuscript, the following suggestions are provided.

**Suggestions:**

 The topic is relevant and falls within the scope of fixed-point theory, and the results may be of interest to researchers working in generalized metric structures and nonlinear analysis.

**Grammatical/Typo:**

 Some suggestions related to grammar and typographical corrections are listed below. The authors are encouraged to carefully review the entire manuscript and implement similar corrections wherever necessary to improve the overall presentation.

 1 In Definition 2.9 add the abbreviation for "occasionally weakly compatible" (O.W.C.)

 2 In Definition 2.7 correct the font of "x"

 3 In Definition 2.13 replace "are called gratify property of

 common limit range related" with "have common limit range property wrt"

 4 In Remark 3.1 is the order of the pairs of three mappings have some significance? if not then just use "are self-mappings".

**Addition of Illustrative Examples:**

 The authors are encouraged to include some illustrative examples to verify the fixed-point results established in the main section of the manuscript. 

**Addition of a Remark or Section:**

 The authors are encouraged to add a remark or a separate Section (or in the Conclusion section) highlighting the results that are extended by the main theorem of the manuscript. If possible, the authors should clearly indicate the earlier results that are generalized by their work, along with appropriate citations to the related literature. For example, a statement such as *“Our result extends Theorem XX of [\cite].”* may be included where applicable.

Is the work clearly and accurately presented and does it cite the current literature?

Yes

If applicable, is the statistical analysis and its interpretation appropriate?

I cannot comment. A qualified statistician is required.

Are all the source data underlying the results available to ensure full reproducibility?

No source data required

Is the study design appropriate and is the work technically sound?

Yes

Are the conclusions drawn adequately supported by the results?

Partly

Are sufficient details of methods and analysis provided to allow replication by others?

Yes

Reviewer Expertise:

Fixed Point Theory of Contraction Mappings, Generalized Metric Structure, Stability of Functional Equations

I confirm that I have read this submission and believe that I have an appropriate level of expertise to confirm that it is of an acceptable scientific standard, however I have significant reservations, as outlined above.

# Reviewer report: Reviewer response for version 2

**DOI:** 10.5256/f1000research.195954.r456310

**Published:** 2026-02-16

**Authors:** Yousif Yaqoub Yousif

**Affiliations:** 1 University of Baghdad, Baghdad, Iraq

The paper can now be accepted for indexing.

Is the work clearly and accurately presented and does it cite the current literature?

Yes

If applicable, is the statistical analysis and its interpretation appropriate?

Yes

Are all the source data underlying the results available to ensure full reproducibility?

Yes

Is the study design appropriate and is the work technically sound?

Yes

Are the conclusions drawn adequately supported by the results?

Yes

Are sufficient details of methods and analysis provided to allow replication by others?

Yes

Reviewer Expertise:

NA

I confirm that I have read this submission and believe that I have an appropriate level of expertise to confirm that it is of an acceptable scientific standard.

# Reviewer report: Reviewer response for version 2

**DOI:** 10.5256/f1000research.195954.r456308

**Published:** 2026-02-10

**Authors:** Khairul Habib Alam

**Affiliations:** 1 Mathematics, Indian Institute of Science Education and Research Berhampur (Ringgold ID: 486382), Brahmapur, Odisha, India

I am satisfied with this version. Kindly cite the following papers for improving the literature. (refer to 1,2,3,4)

Is the work clearly and accurately presented and does it cite the current literature?

Partly

If applicable, is the statistical analysis and its interpretation appropriate?

I cannot comment. A qualified statistician is required.

Are all the source data underlying the results available to ensure full reproducibility?

Partly

Is the study design appropriate and is the work technically sound?

Partly

Are the conclusions drawn adequately supported by the results?

Yes

Are sufficient details of methods and analysis provided to allow replication by others?

Yes

Reviewer Expertise:

Fixed point theory, generalized metric structures, iterative algorithms, and applied optimization

I confirm that I have read this submission and believe that I have an appropriate level of expertise to confirm that it is of an acceptable scientific standard.

## References

[1] : Fixed point and its geometry and application for multivalued integral type contractions in $m_v^b$-metric spaces. *Hacettepe Journal of Mathematics and Statistics* .2025;54(5) : 10.15672/hujms.1471688 1708-1724 10.15672/hujms.1471688

[2] : Set-valued contractions with an application to Fredholm integral inclusions in $ {m_v^b}- $metric spaces. *AIMS Mathematics* .2025;10(9) : 10.3934/math.2025926 20742-20758 10.3934/math.2025926

[3] : A new extended metric space and solution of rocket’s ascending motion. *Asian-European Journal of Mathematics* .2025;18(06) : 10.1142/S1793557125500019 10.1142/S1793557125500019

[4] : On geometry of fixed figures via φ−interpolative contractions and application of activation functions in neural networks and machine learning models. *Ain Shams Engineering Journal* .2025;16(1) : 10.1016/j.asej.2024.103182 10.1016/j.asej.2024.103182

# Reviewer report: Reviewer response for version 1

**DOI:** 10.5256/f1000research.189957.r441497

**Published:** 2026-01-10

**Authors:** Khairul Habib Alam

**Affiliations:** 1 Mathematics, Indian Institute of Science Education and Research Berhampur (Ringgold ID: 486382), Brahmapur, Odisha, India

**Peer Review Report**

 Article Title: Some Extended Results of Common Fixed Point Theorems via Enhanced Categories of Contractive Mappings in D_d*-Symmetric Spaces

 The manuscript investigates standard fixed point theorems for three pairs of self-mappings within the framework of D_d*-Symmetric Spaces. The authors utilize the property of Common Limit in the Range (CLR) and the concept of Occasionally Weakly Compatible (OWC) mappings to establish unique common fixed point results.

- **Language and Grammar:** The manuscript suffers from significant grammatical errors, awkward phrasing, and sentence structure issues that impede readability. A thorough proofreading is required.
- **Literature Review:** While the historical context is provided, the bibliography lacks recent developments in related generalized metric spaces (specifically, 2025 advancements) and applications to differential/integral inclusions, which the authors claim their work supports.
- **Clarity of Definitions:** Some definitions are verbose and could be streamlined for better mathematical precision.

**Grammatical Errors and Sentence Clarity**

 The English usage throughout the manuscript needs substantial polishing. Below are specific examples of errors and suggested corrections.

**Current Text:** "The Banach fixed point theory stated that a mapping T: X to X always has a unique fixed point..."

**Correction:** "Banach fixed point theory **states** that..."

**Current Text:** "Various vital concepts such as (D_d*-Symmetric and weakly compatible maps) that are needed in the sequel, which will help us in the outcomes that follow and play a major role in verifying our major outcomes."

**Correction:** "Vital concepts, such as D_d*-Symmetric Spaces and weakly compatible maps, are reviewed to establish the framework for our main results."

**Current Text:** "Our main results in this manuscript have explored novel various outcomes..."

**Correction:** "This manuscript explores novel outcomes regarding the uniqueness of..."

**Current Text:** "...various extensions of Banach fixed point theory were carried out. Therefore, fixed point theory has been developed and diversified..."

**Correction:** "...various extensions of Banach fixed point theory were established. Consequently, the theory has evolved to encompass diverse extensions..."

**Current Text:** "...talented with partial ordering."

**Correction:** "... **endowed** with a partial ordering."

**Current Text:** "Our motivation for introducing this segment is to study and verify..."

**Correction:** "In this section, we establish the uniqueness..."

**Current Text:** "Let S, Q, F, F, G and J be three pairs of self-maps..."

**Correction:** "Let S, Q, F, G, and J **be** three pairs of self-maps..."

**Current Text:** "We anticipate that the discoveries in this manuscript will aid scientists in enhancing the authors' understanding of popularized extended symmetric spaces..."

**Correction:** "We anticipate that these results will aid researchers in developing generalized symmetric spaces and establishing frameworks for applications in advanced sciences."

**Improvement of Literature and Citations**

 The Introduction and Conclusion claim that fixed point theory is vital for finding solutions to "linear and nonlinear systems," "integral and differential equations," and "optimization theory." However, the current references do not sufficiently reflect the most recent advancements in these specific applications (particularly in 2025).

 To substantiate the paper's claims regarding applicability and to situate the work within the state-of-the-art, **the following recent papers must be cited.** These works deal with similar generalizations (M_v^b-metric spaces, fuzzy spaces) and explicitly demonstrate the applications (Fredholm integrals, differential inclusions) that the authors allude to.

**Suggested Additions:**

1. **Context:** When discussing applications to integral equations and set-valued mappings (e.g., Introduction, paragraph 1 or 5), cite:

*https://doi.org/10.15672/hujms.1471688*

1. **Context:** When mentioning extensions to fuzzy structures or applications to nonlinear Cauchy differential inclusions (e.g., Introduction, near the discussion of metric space generalizations), cite:

*https://doi.org/10.1177/18758967251366331*

1. **Context:** When discussing contraction principles and their application to Fredholm integral inclusions (specifically to support the claim in the "Background" section about integral equations), cite:

*https://doi.org/10.3934/math.2025926*

1. **Context:** When referring to "optimization theory" or proximal contractions (e.g., Introduction or Conclusion), cite:

*https://doi.org/10.1186/s13663-025-00799-0*

 Integration Strategy:

 The authors should add a paragraph in the Introduction (around the discussion of "Promised studies" or "Motivated by above facts") that reads:

*"Recent developments in 2025 have further expanded fixed point theory into complex generalized spaces with robust applications. For instance, new results in M_v^b-metric spaces have addressed multivalued integral contractions [1] and Fredholm integral inclusions [3]. Furthermore, significant advances have been made in the application of fuzzy metric spaces to nonlinear differential inclusions [2] and proximal contractions to optimization problems [4]. These works highlight the continued relevance of extending contractive conditions in generalized spaces."*

**Other Comments**

- Symmetry of D_d*: In the proofs (e.g., Theorem 3.2), the authors frequently state "because D_d* is symmetric." It would be beneficial to explicitly reference the specific property number from Definition 2.1 (e.g., $D_3$) to assist the reader.
- **Inequality Numbering:** Ensure that the labeling of inequalities (1), (2), (3) is consistent and that they are referenced correctly throughout the text.
- **Uniqueness Proof:** The uniqueness proof in Theorem 3.2 is generally sound, but the notation for the arbitrary fixed points (neq t) should be defined clearly at the start of the uniqueness paragraph.
- **Examples:** Authors have to add numerical examples to validate the results.
- **Graphical Representation:** Authors can add a graphical representation in the numerical examples.
- **Application:** The Author can add an application to the real-world problem.

**Decision**

 The paper presents interesting results in the domain of symmetric spaces. However, the presentation is currently not up to the standard of a high-impact journal. I recommend **Major Revisions**.

Is the work clearly and accurately presented and does it cite the current literature?

Partly

If applicable, is the statistical analysis and its interpretation appropriate?

I cannot comment. A qualified statistician is required.

Are all the source data underlying the results available to ensure full reproducibility?

Partly

Is the study design appropriate and is the work technically sound?

Partly

Are the conclusions drawn adequately supported by the results?

Yes

Are sufficient details of methods and analysis provided to allow replication by others?

Yes

Reviewer Expertise:

Fixed point theory, generalized metric structures, iterative algorithms, and applied optimization

I confirm that I have read this submission and believe that I have an appropriate level of expertise to confirm that it is of an acceptable scientific standard, however I have significant reservations, as outlined above.

# Response

**Published:** 2026-01-26

**Authors:** Alaa AL-Jumaili

**Affiliations:** Department of mathematics, College of Education for pure sciences, University of Anbar, Ramadi, Alanbar, Iraq

Report and Comments responses-f1000res172242

 Dear prof...

 Best greetings.....

 I hope this message finds you well.

 Subject : comments responses about the manuscript-f1000res172242

  (Some Extended Results of Common Fixed Point Theorems via Enhanced Categories of Contractive Mappings in Dd*- Symmetric Spaces)-Manuscript Number: 3b619270-4ddc-4685-af42-071e2dbdcc63-f1000res172242)

 First: We extend our sincere thanks to the esteemed evaluator for what he said regarding the paper being good and its results being original, as well as the originality of the examples and applications presented in it.           

 The following is the response to the comments kindly provided by the esteemed resident, and they have been carefully considered due to their importance.

 1- The manuscript has already been carefully reviewed for linguistic and spelling accuracy in all its parts.

 2- Some lengthy definitions have been simplified to improve their mathematical precision such as (Definition 2.3 and 2. 8).

 3- All the errors pointed out by the esteemed evaluator have been corrected by implementing all his valuable suggestions.

 4- All the references [27-30] which suggested by the esteemed evaluator have been added due to their importance in raising the scientific level for out paper and rigor of the manuscript y and to situate out work within the state-of-the-art.

 5- We confirm the addition of the paragraph proposed by the honored evaluator to the introduction (around the discussion of Promised studies or Motivated).

 6- It was explicitly stated reference the specific property number from Definition 2.1 in all parts in the paper

 7- We would like to assure that all of labeling of inequalities (1), (2) and (3) are consistent and they are referenced correctly throughout the text.

 8- We would like to assure you that all of notations for the arbitrary fixed points (C.F.P) have been defined clearly at the start of the uniqueness paragraph in the proof in Theorem 3.2.

 9- We anticipate that these results will aid researchers in developing generalized symmetric spaces and establishing frameworks for applications in advanced sciences.

 Acknowledgments: thankful to reviewers for their valuable corrections and important suggestions  

# Reviewer report: Reviewer response for version 1

**DOI:** 10.5256/f1000research.189957.r441499

**Published:** 2026-01-05

**Authors:** Yousif Yaqoub Yousif

**Affiliations:** 1 University of Baghdad, Baghdad, Iraq

**I would like to inform you that several comments have been registered about the following paper:**

**(** **Some Extended Results** **of Common Fixed Point Theorems via Enhanced Categories of Contractive Mappings in** **D** **d** *  **- Symmetric Spaces** **)-Manuscript Number: 3b619270-4ddc-4685-af42-071e2dbdcc63-f1000res172242)**

1. **General Comments:**  In this manuscript, some new generalized common fixed point theorems for three pairs of self-maps under the influence of other enhanced categories of extended contractive conditions in the context of D *d* *  -Symmetric spaces have been presented and verified.  Also, by applying the concepts of weak compatibility and common limit in the range property some main results have been established.
2. The presentation method of the abstract is a bit disorganized and I believe it needs some expansion and focus on the main objectives of the manuscript.
3. Please, state the most important results obtained at the final of the introduction so as to display the latest results obtained compared to previous studies.
4. I think that the first condition in definition (2. 1) is extra. It is evident. Remove it
5. Could you please add the Lemma in the reference [27] in the last of Remark 2.4 to the Fundamentals chapter?
6. Please, could you cite the reference for the remark (3. 1) observation into the main results chapter?
7. Could you please extend the proof of the theorem (3. 2) to be easier for authors to follow?
8. Why wasn't the proof for the Corollary (3.3) mentioned in detail?
9. Please check all punctuation marks in the all sections in the manuscript.
10. The authors should be replacing the word (gratify) by (satisfy) and the symbol (&) by (and) in all sections of their manuscript.
11. I think contributions and research objectives/questions are not clearly presented.
12. Are there any practical applications for this type of research? Please explain them.

 With kind regards,

 Yours sincerely****

 Prof. Dr. Yousif Yaqoub Yousif

 University of Baghdad

 College of Education For Pure science (Ibn Al-Haitham)

 Department of Mathematics – Baghdad – Iraq

 Mathematics – Topology and its Applications

 E-mail : yousif.y.y@ihcoedu.uobaghdad.edu.iq

**Scopes :** https://www.scopus.com/authid/detail.uri?authorId=54790328600

**ORCID :** https://orcid.org/0000-0002-6834-3515 https://orcid.org/0000-0002-6834-3515

**Google Scholar :** https://scholar.google.com/citations?hl=en&user=Eax_5YIAAAAJ

**Research Gate :** https://www.researchgate.net/profile/Yousif_Yousif3

Is the work clearly and accurately presented and does it cite the current literature?

Yes

If applicable, is the statistical analysis and its interpretation appropriate?

Yes

Are all the source data underlying the results available to ensure full reproducibility?

Yes

Is the study design appropriate and is the work technically sound?

Yes

Are the conclusions drawn adequately supported by the results?

Yes

Are sufficient details of methods and analysis provided to allow replication by others?

Yes

Reviewer Expertise:

NA

I confirm that I have read this submission and believe that I have an appropriate level of expertise to confirm that it is of an acceptable scientific standard, however I have significant reservations, as outlined above.

# Response

**Published:** 2026-01-06

**Authors:** Alaa AL-Jumaili

**Affiliations:** Department of mathematics, College of Education for pure sciences, University of Anbar, Ramadi, Alanbar, Iraq

**Dear Editor...**

**Best greetings.....**

**I hope this message finds you well.**

**Subject \ comments responses about the manuscript -f1000res172242**

**(** **Some Extended Results** **of Common Fixed Point Theorems via Enhanced Categories of Contractive Mappings in** **D** **d** * **- Symmetric Spaces** **)-Manuscript Number: 3b619270-4ddc-4685-af42-071e2dbdcc63-f1000res172242)**

**First: We extend our sincere thanks to the esteemed evaluator for what he said regarding the paper being good and its results being original, as well as the originality of the examples and applications presented in it The following is the response to the comments kindly provided by the esteemed resident, and they have been carefully considered due to their importance.**

**1-** **Answer** **:** **Yes, This represents an abstract of the main objectives of our manuscript** **.**

**2-** **Answer** **:** **The main objectives of our manuscript are highlighted in the abstract and cannot be expanded further because the journal has specified the word count for the abstract, and it has also been formatted for optimal clarity.**

**3-** **Answer** **:** **The main findings of this paper are listed at the end of the introduction, as directed by the respected reviewer.**

**4-** **Answer** **:** **The first condition in definition (2. 1) is essential condition and it is found in the original paper [26].**

**5-** **Answer** **:** **The Lemma which was mentioned at the end of Remark (2.4) in our manuscript is already present in reference [27] and any author can View it.**

**6-** **Answer** **:** **The remark (3. 1) is from our presenting and has been included because we needed it in our main results.**

**7-** **Answer** **:** **I think that the proof of Theorem No. (3. 2) is easy and clear and can be easily followed by researchers.**

**8- Answer** **:** **Because the Corollary (3.3) is a direct result of Theorem (3. 2) and its proof follows immediately from Theorem-3.2, via putting** **G** **=** **F** **=** **I** **X**  **(Identity mapping).**

**9- Answer** **:** **All punctuation marks in the all sections of the manuscript have been checked and verified as directed by the respected reviewer.**

**10- Answer** **:** **The term "gratify" and the symbol (**& **) will be replaced in all chapters of the paper in the final version.**

**11- Answer** **:** **Our contributions and research objectives have been presented in the end of the introduction.**

**12- Answer** **:** **Yes, there are certainly many any practical applications for the topic of this paper in many fields of mathematics and other branches of science, such as engineering, physics, computer sciences, economics, and telecommunication optimization problems.**

**13- Answer** **:** **Yes, as it is known to all scientists and researchers, every metric space is T2 a topological space, as shown in the link attached below** **.**

**https://math.uchicago.edu/~may/REU2013/REUPapers/Clarke.pdf**

**14 Answer** **:** **The applications of fixed point theory in the fields of mathematics and other branches of sciences have demonstrated in the introduction part.**

**15- Answer** **:** **All names of journals in the source list have been checked and verified as directed by the respected reviewer.**

# Reviewer report: Reviewer response for version 1

**DOI:** 10.5256/f1000research.189957.r441496

**Published:** 2026-01-02

**Authors:** Choonkil Park

**Affiliations:** 1 Hanyang University, Seoul, South Korea

The results are interesting. But the present form is not readable. The authors did not read the final version before submitting the paper. For example, (1) Definition 2.1 [26] A ; (2) Example 2.2 [26] Assume that ; Theorem 3.5 If ~ are six self-maps in. The paper should be revised to be reviewed again.  The present form contains a lot of errors in typos and grammatical errors.

 The paper should be revised to be reviewed again.

Is the work clearly and accurately presented and does it cite the current literature?

Partly

If applicable, is the statistical analysis and its interpretation appropriate?

Partly

Are all the source data underlying the results available to ensure full reproducibility?

Partly

Is the study design appropriate and is the work technically sound?

Yes

Are the conclusions drawn adequately supported by the results?

Partly

Are sufficient details of methods and analysis provided to allow replication by others?

Yes

Reviewer Expertise:

Fixed point theory, Functional analysis, Nonlinear analysis, Fuzzy analysis

I confirm that I have read this submission and believe that I have an appropriate level of expertise to state that I do not consider it to be of an acceptable scientific standard, for reasons outlined above.

# Response

**Published:** 2026-01-12

**Authors:** Alaa AL-Jumaili

**Affiliations:** Department of mathematics, College of Education for pure sciences, University of Anbar, Ramadi, Alanbar, Iraq

First: We extend our sincere thanks to the esteemed evaluator for what he said regarding the paper being good and its results being original, as well as the originality of the examples and applications presented in it.

 The following is the response to the comments kindly provided by the esteemed resident, and they have been carefully considered due to their importance.

- We extend our deep thanks to the honored reviewer for his valuable observations, which aim to improve the research paper in order to enhance its rigor and scientific value and avoid typographical and grammatical errors. We would like to clarify the following:

**1** **-**We would like to assure you that the manuscript was thoroughly reviewed before submission, but some typographical errors may have occurred during reprinting and were not fully noticed. These will be corrected in the final version before submission.

**2** **-**We would like to assure you that the manuscript has been linguistically revised by the esteemed journal, and we confirm that all the linguistic revisions sent by the journal have been taken into account due to their great importance to this research paper.

**3** **- **Also, we would like to assure you that the research paper will be thoroughly reviewed before the final version is sent, as per the esteemed reviewer's instructions.
