# Supplementary material for: Some Extended Results of Common Fixed Point Theorems via Enhanced Categories of Contractive Mappings in Dd* - Symmetric Spaces
Source: F1000Res. 2025 Dec 5;14:1363. [Version 1] doi: 10.12688/f1000research.172242.1 (PMC13142793; doi:10.12688/f1000research.172242.1)
Supplement: Peer review history

# Response

**Published:** 2026-01-12

**Authors:** Alaa AL-Jumaili

**Affiliations:** Department of mathematics, College of Education for pure sciences, University of Anbar, Ramadi, Alanbar, Iraq

First: We extend our sincere thanks to the esteemed evaluator for what he said regarding the paper being good and its results being original, as well as the originality of the examples and applications presented in it.                                                                 

 The following is the response to the comments kindly provided by the esteemed resident, and they have been carefully considered due to their importance.

- We extend our deep thanks to the honored reviewer for his valuable observations, which aim to improve the research paper in order to enhance its rigor and scientific value and avoid typographical and grammatical errors. We would like to clarify the following:

**1** **-**We would like to assure you that the manuscript was thoroughly reviewed before submission, but some typographical errors may have occurred during reprinting and were not fully noticed. These will be corrected in the final version before submission.

**2** **-**We would like to assure you that the manuscript has been linguistically revised by the esteemed journal, and we confirm that all the linguistic revisions sent by the journal have been taken into account due to their great importance to this research paper.

**3** **- **Also, we would like to assure you that the research paper will be thoroughly reviewed before the final version is sent, as per the esteemed reviewer's instructions.
